# Phenomenology of Anorexia Nervosa in Cambodia: A Transcultural Approach

**DOI:** 10.1192/j.eurpsy.2025.1935

**Published:** 2025-08-26

**Authors:** S. Bora, R. Ngin, S. Vilhem

**Affiliations:** 1 Cambodian Children’s Fund, Phnom Penh, Cambodia; 2 University of Lausanne, Lausanne, Switzerland

## Abstract

**Introduction:**

Anorexia Nervosa (AN) is a complex psychiatric disorder often understood through a Western lens. This study explores AN in the Cambodian context, where the condition is not traditionally recognized, to uncover cultural and transcultural aspects of the illness.

**Objectives:**

This research aims to conduct an in-depth phenomenological exploration of AN cases in Cambodia, documenting the lived experiences of individuals and identifying cultural or transcultural elements within the universal description of AN.

**Methods:**

Employing a qualitative research approach grounded in phenomenology, this study will involve in-depth interviews with Cambodian individuals experiencing AN. The data will be analyzed using both descriptive and interpretive phenomenological methods, ensuring the bracketing of researcher bias and fostering co-creation of interpretations.

**Results:**

Expected outcomes include a comprehensive phenomenological account of AN in Cambodia, shedding light on the lived experiences of individuals and potentially revealing unique cultural dimensions of the disorder. The study will contribute to the understanding of AN from a transcultural perspective, highlighting both universal and culturally specific aspects.

**Conclusions:**

This research is anticipated to provide valuable insights into the phenomenology of AN in a non-Western context. The findings may have implications for clinical practice, research, and cross-cultural understanding of AN, emphasizing the importance of patient-centered approaches and the exploration of lived experiences in psychopathology.

**Disclosure of Interest:**

S. Bora Grant / Research support from: International Exchange Award from the Renewing Phenomenological Psychopathology funded by the Wellcome Trust, R. Ngin: None Declared, S. Vilhem: None Declared

